# A Novel Tool for the Generation of Conditional Knockouts To Study Gene Function across the Plasmodium falciparum Life Cycle

**DOI:** 10.1128/mBio.01170-19

**Published:** 2019-09-17

**Authors:** Marta Tibúrcio, Annie S. P. Yang, Kazuhide Yahata, Pablo Suárez-Cortés, Hugo Belda, Sebastian Baumgarten, Marga van de Vegte-Bolmer, Geert-Jan van Gemert, Youri van Waardenburg, Elena A. Levashina, Robert W. Sauerwein, Moritz Treeck

**Affiliations:** a Signalling in Apicomplexan Parasites Laboratory, The Francis Crick Institute, London, United Kingdom; b Department of Medical Microbiology, Radboud University Medical Centre, Nijmegen, Netherlands; c Department of Protozoology, Institute of Tropical Medicine (NEKKEN), Nagasaki University, Nagasaki, Japan; d Biology of Host-Parasite Interactions Unit, Institut Pasteur, Paris, France; e Vector Biology Unit, Max Planck Institute for Infection Biology, Berlin, Germany; NIAID/NIH

**Keywords:** *Plasmodium falciparum*, malaria, molecular methods, reverse genetic analysis

## Abstract

Plasmodium falciparum has a complex life cycle that involves interaction with multiple tissues inside the human and mosquito hosts. Identification of essential genes at all different stages of the P. falciparum life cycle is urgently required for clinical development of tools for malaria control and eradication. However, the study of P. falciparum is limited by the inability to genetically modify the parasite throughout its life cycle with the currently available genetic tools. Here, we describe the detailed characterization of a new marker-free P. falciparum parasite line that expresses rapamycin-inducible Cre recombinase across the full life cycle. Using this parasite line, we were able to conditionally delete the essential invasion ligand AMA1 in three different developmental stages for the first time. We further confirm efficient gene deletion by targeting the nonessential kinase FIKK7.1.

## Abstract (precis)

One of the major limitations in studying P. falciparum is that so far only asexual stages are amenable to rapid conditional genetic modification. The most promising drug targets and vaccine candidates, however, have been refractory to genetic modification because they are essential during the blood stage or for transmission in the mosquito vector. This leaves a major gap in our understanding of parasite proteins in most life cycle stages and hinders genetic validation of drug and vaccine targets. Here, we describe a method that supports conditional gene deletion across the P. falciparum life cycle for the first time. We demonstrate its potential by deleting essential and nonessential genes at different parasite stages, which opens up completely new avenues for the study of malaria and drug development. It may also allow the realization of novel vaccination strategies using attenuated parasites.

## INTRODUCTION

Malaria is one of the world’s deadliest diseases, causing 219 million cases worldwide in 2017 and 435,000 deaths. Plasmodium falciparum is responsible for >95% of the reported cases (1). One of the greatest threats to malaria control is the emerging resistance to all frontline drugs and the limited protection of the only approved vaccine. Therefore, the development of novel drugs and vaccines becomes imperative and urgent. A better understanding of the malaria parasite biology at each developmental stage is fundamental and needed to accomplish this goal. This will lead to effective novel malaria interventions, which should target several of the parasite stages in the human host, and many drug screens are targeted toward multistage activity (2, 3). *Plasmodium* parasites are transmitted by blood-feeding infected mosquitoes that inject sporozoites into the human host. Sporozoites travel to the liver and invade hepatocytes where they develop into thousands of merozoites that will be released back into circulation. Once merozoites are in the bloodstream, they invade erythrocytes and can either develop through a 48-h asexual cycle or develop sexually. The sexual forms, the gametocytes, are the only stages that can mediate transmission through the mosquito. Inside the mosquito, the gametocytes are activated, and fertilization occurs, resulting in the formation of motile ookinetes that develop into oocysts, which then develop into thousands of infective sporozoites.

One of the major bottlenecks when studying P. falciparum is the limited ability to genetically modify the parasite in most life cycle stages, as the genetic tools for identification of essential genes across the P. falciparum life cycle are nonexistent (4). As *Plasmodium* species are haploid during the asexual parasite stage when genetic modifications are feasible, essential genes can be targeted only by conditional systems. Several of these systems have been developed to allow downregulation of mRNA levels (5–7), translation (8), proteins (9), or protein mislocalization (10). While the RNA and protein regulation systems allow reversible control of protein levels, they can suffer from background protein levels and require constant drug pressure. Conditional gene deletions can be achieved using the FLP-frt system which has been developed to conditionally test gene function during transmission in Plasmodium berghei and has been adapted to P. falciparum (11). However, the requirement for stage-specific promoters to drive recombinase activity at different life cycle stages severely limits its usability in P. falciparum. A more reliable gene inactivation method is the rapamycin-induced activation of dimerizable Cre recombinase (12). In combination with loxP sites in artificial introns (loxPint), it allows rapid generation of floxed genetic elements for gene truncations, deletions, domain replacements, and conditional introduction of point mutations (13). All of these methods have been useful to study genes in the erythrocytic stages of P. falciparum, but not for genes important for mosquito transmission or liver stages, where such systems are highly needed.

In this study, we describe a novel parasite line that supports conditional gene deletion in three different developmental stages and the study of essential genes across the P. falciparum life cycle. This novel tool and approach can therefore be used to investigate P. falciparum genes in most life cycle stages and genetically validate novel multistage drug and vaccine candidates

## RESULTS

### Characterization of an NF54::DiCre line across the P. falciparum life cycle.

**FIG 1:**
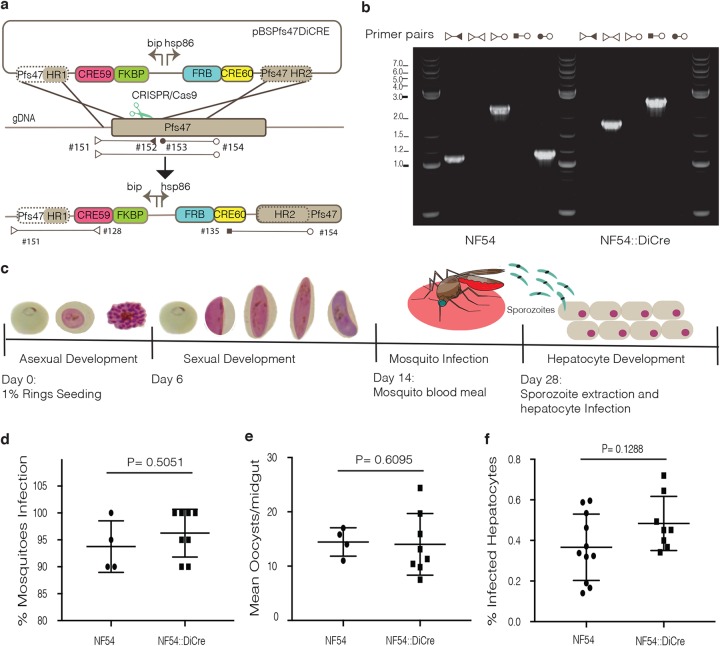
Generation and characterization of the NF54::DiCre line for conditional gene deletions across the P. falciparum life cycle. (a) Schematics of the targeting (pBSPs47DiCre) plasmid, the expected recombination product, and representation of the primer pairs used to test integration. (b) PCR analysis of DiCre cassette integration into the Pfs47 locus in P. falciparum NF54. The sequences of the primers used (e.g., primer #151) are shown in Table S1 in the supplemental material. (c) Illustration of the P. falciparum life cycle and expected timeline of the different developmental stages addressed in this paper. (d) Percentage of A. stephensi mosquitoes infected with NF54 and NF54::DiCre after a blood meal, where each data point corresponds to the value from an independent experiment. (e) Number of NF54 and NF54::DiCre oocysts per mosquito gut. Each data point corresponds to the value from an independent experiment. (f) Comparison of hepatocyte infection rates between NF54 and NF54::DiCre parasites of at least four independent experiments, each with two technical replicates. All *P* values were calculated by the Mann-Whitney *t* test.

Rapamycin-induced activation of dimerized Cre recombinase has proven highly efficient in P. falciparum (12, 14–17). To enable conditional gene deletions at different time points across the life cycle, we introduced a DiCre cassette into the *pfs47* locus of NF54 parasites using CRISPR/Cas9, resulting in a marker-free NF54::DiCre parasite line (Fig. 1a and b). Pfs47 was previously reported to play an important role during transmission in Anopheles gambiae but not in Anopheles stephensi (18). We first tested the NF54::DiCre ability to develop across the P. falciparum life cycle (Fig. 1c). As expected, the NF54::DiCre line shows no differences in growth compared to the parental line during asexual and sexual development (data not shown) or infection of A. stephensi mosquitoes (Fig. 1d and e). Moreover, sporozoites show normal invasion rates and development in primary hepatocytes (Fig. 1f). To exclude any loss of genes caused by extended periods of time in cell culture (19), we sequenced the genome of NF54::DiCre (BioProject accession no. PRJNA422809) and found no differences from an NF54 isolate from a different laboratory (see Fig. S1 in the supplemental material). These data suggest that the NF54::DiCre parasite line can be efficiently used as a background to conditionally delete genes across the life cycle.

10.1128/mBio.01170-19.1 FIG S1 Whole-genome alignment of the P. falciparum NF54 DiCre nuclear genome reported in this study and the wild-type P. falciparum 3D7 and NF54 nuclear genome. Red lines represent high-similarity alignments (≥99%) with red dots indicating start/end points. The final nuclear assembly is composed of 22 contigs with a total size of 23,440,593 bases and an average GC content of 19.36%. All 14 chromosomes present in the 3D7 and NF54 strains are found in the NF54 DiCre assembly, with eight contigs representing putatively full-length chromosomes. The remaining six chromosomes are assembled in no more than three contigs per chromosome. In total, 5,592 loci were identified in the nuclear genome, of which 5,061 are protein-coding genes, 357 are putative pseudogenes, and 174 are non-protein-coding RNA. Download FIG S1, TIF file, 0.8 MB. Copyright © 2019 Tibúrcio et al. 2019 Tibúrcio et al. This content is distributed under the terms of the Creative Commons Attribution 4.0 International license.

10.1128/mBio.01170-19.7 TABLE S1 Primers used to confirm integration and efficient rapamycin-induced excision of pBSPs47DiCre, FIKK7.1:loxPint:HA, and Ama1:loxPint:HA plasmids. Download Table S1, DOCX file, 0.02 MB. Copyright © 2019 Tibúrcio et al. 2019 Tibúrcio et al. This content is distributed under the terms of the Creative Commons Attribution 4.0 International license.

### Rapamycin-mediated conditional truncation of AMA1 in asexual, gametocyte, and hepatocyte stages.

**FIG 2:**
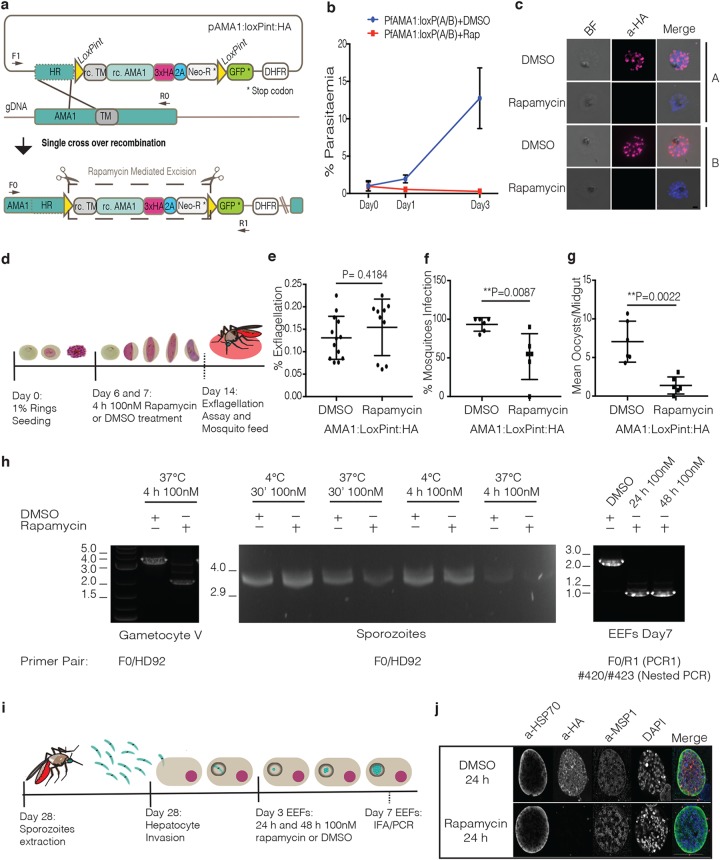
Characterization of rapamycin-mediated excision efficiency of the AMA1 gene in the NF54::DiCre line across P. falciparum life cycle. (a) Description of the strategy used to make the AMA1 conditional knockout (KO) line (AMA1:loxPint:HA) and the primers used to confirm integration. The rescue plasmid contains a recodonized version of the C-terminal *ama1* gene (rc. AMA1), followed by a triple-HA (3xHA), T2A peptide (2A), and neomycin resistance cassette (Neo-R), flanked by loxPints. A GFP cassette is used to monitor rapamycin-mediated excision events. (b) Growth curve comparing DMSO- and rapamycin-treated asexual parasites of two independent transfections (populations A and B) representing two independent experiments. (c) Immunofluorescence analysis of DMSO- and rapamycin-treated asexual parasites from populations A and B using anti-HA antibody (α-HA) to check for efficient rapamycin-induced excision. (d) Representation of the experimental workflow to test rapamycin-induced AMA1 KO during early sexual development. (e) Exflagellation assays comparing male gametocyte exflagellation centers of DMSO- versus rapamycin-treated gametocytes (percent total mature gametocytes). The data points represent the values for technical replicates from two independent experiments. (f) A. stephensi infection rates comparing DMSO- versus rapamycin-treated AMA1:loxPint:HA gametocytes. Each data point corresponds to the value of an independent experiment. (g) Average number of oocysts per mosquito midgut from DMSO- and rapamycin-treated AMA1:loxPint:HA parasites. Each data point corresponds to the value of an independent experiment. (h) PCR analysis comparing DMSO- versus rapamycin-treated parasite genomic DNA extracted from different developmental stages. Gametocytes were treated sequentially on days 6 and 7 after induction, sporozoites were treated either for 30 min (30’) or 4 hours (4h) at either 4°C or 37°C, and EEFs were treated for 24 h or 48 h. The sequences of the primers used are shown in Table S1. (i) Description of DMSO and rapamycin treatment point for conditional gene deletion during hepatocyte development. (j) Immunofluorescence analysis of EEFs on day 7 after hepatocyte infection comparing DMSO versus rapamycin treatment. All *P* values were calculated by the Mann-Whitney *t* test.

To test the recombination efficiency of the NF54::DiCre line at different developmental stages, we generated a conditional knockout (KO) of the blood-stage essential apical membrane antigen 1 (*ama1*) gene in the NF54::DiCre line. AMA1 has been well characterized during asexual development in P. falciparum (20, 21), but its role during transmission and hepatocyte invasion in different species is less well understood (22–24). We inserted loxP sites in artificial introns (13) upstream of the AMA1 transmembrane domain (TM) and downstream of its cytoplasmic tail, resulting in a hemagglutinin (HA)-tagged AMA1 protein predicted to produce a soluble AMA1 isoform upon rapamycin treatment (20) (Fig. 2a). As expected, treatment of asexual stages with rapamycin leads to truncation of *ama1* and an inability to grow (Fig. 2b). Efficient loss of the HA tag was verified by immunofluorescence assay, PCR, and Western blotting (Fig. 2c and Fig. S2 and S3). Having confirmed that deletion of the AMA1 C terminus inactivates AMA1, we induced gene excision during sexual development by treating the parasites with rapamycin on days 6 and 7 after induction (Fig. 2d). Phenotypic analysis of sexual development revealed no significant difference in development, exflagellation, or macrogamete formation rates between dimethyl sulfoxide (DMSO)- and rapamycin-treated cultures (Fig. 2e and Fig. S4). The mature sexual cultures were then fed to the A. stephensi mosquitoes. We observed a significant reduction of infected mosquitoes (43%) as well as a substantial reduction of oocyst numbers (82.4%) in rapamycin-treated parasites compared with the DMSO control (Fig. 2f and g). Analysis of the few resulting sporozoites for *ama1* deletion efficiency by immunofluorescence revealed that 75% of all parasites showed loss of HA signal, despite near complete excision of the locus during sexual stages, indicating some positive selection for parasites during transmission that have functional AMA1 (Fig. 2h and Fig. S5). Unfortunately, the low numbers of sporozoites obtained from the rapamycin-treated gametocyte cultures prevented us from performing hepatocyte invasion assays with AMA1 mutants. However, because the DMSO-treated parasites transmitted well, we attempted to induce AMA1 truncations of isolated sporozoites with rapamycin using different concentrations and incubation times (Fig. 2h). None of the conditions tested resulted in recombination of the *ama1* locus (Fig. 2h). We then tested the possibility of conditionally deleting *ama1* during liver-stage development after sporozoite invasion (Fig. 2i). At 4 h postinfection (hpi), no excision could be detected (data not shown). However, rapamycin treatment at 72 hpi resulted in efficient excision without affecting the development of exoerythrocytic forms (EEFs) development (Fig. 2h and j). Immunofluorescence and PCR analyses showed complete loss of HA signal and excision of the floxed DNA, respectively, in EEFs after 7 days of development (Fig. 2h and j). While further work is required to pinpoint the role of AMA1 during transmission, these data show that efficient rapamycin-mediated gene deletion can be achieved in asexual, sexual, and liver stages using an NF54::DiCre line.

10.1128/mBio.01170-19.2 FIG S2 Confirmation of AMA1:loxPint:HA integration into the NF54::DiCre parasite line and rapamycin excision efficiency. (a) Overview of the strategy used to make a conditional KO by introducing a recodonized version of AMA1 flanked by two loxPints. Representation of the primer pairs used to test correct integration of AMA1:loxPint:HA and efficient rapamycin-mediated excision. (b) PCR analysis of the two independently transfected populations (populations A and B) shows almost complete excision after rapamycin (R) treatment compared with DMSO (D) in asexual stages. P indicates the plasmid pAMA1:loxPint:HA. The sequences of the primers used are shown in Table S1. Download FIG S2, TIF file, 0.5 MB. Copyright © 2019 Tibúrcio et al. 2019 Tibúrcio et al. This content is distributed under the terms of the Creative Commons Attribution 4.0 International license.

10.1128/mBio.01170-19.3 FIG S3 Characterization of AMA1:loxPint:HA protein expression in the presence and absence of rapamycin in asexual parasites. (a) Live-cell imaging of GFP expression in the AMA1:loxPint:HA line after rapamycin-induced gene excision in two independently transfected populations. The live-cell imaging results show GFP expression only in rapamycin-treated asexual parasites. (b) Western blot analysis of DMSO- and rapamycin-treated parasites was done using anti-HA antibody and anti-EBA175 antibody (loading control). The Western blotting (WB) results show that HA is expressed in the DMSO-treated parasites (populations A and B), but almost no HA signal is detected in the rapamycin-treated parasites in the WB. Download FIG S3, TIF file, 1.1 MB. Copyright © 2019 Tibúrcio et al. 2019 Tibúrcio et al. This content is distributed under the terms of the Creative Commons Attribution 4.0 International license.

10.1128/mBio.01170-19.4 FIG S4 Characterization of AMA1 conditional KO line during macrogamete formation. (a) Illustration of the parasite treatment with DMSO/rapamycin on day 6 and 7 during sexual induction and of the macrogamete assay performed on day 15. (b) The results from the macrogamete assay do not show a significant difference in the percentage of female gametes formed (of total mature gametocytes) when comparing DMSO- versus rapamycin-treated parasites. *P* values were calculated by the Mann-Whitney *t* test. Download FIG S4, TIF file, 0.8 MB. Copyright © 2019 Tibúrcio et al. 2019 Tibúrcio et al. This content is distributed under the terms of the Creative Commons Attribution 4.0 International license.

10.1128/mBio.01170-19.5 FIG S5 Characterization of AMA1 protein expression in sporozoites after conditional deletion during sexual stages. (a and b) Illustration of the parasite treatment with DMSO/rapamycin on days 6 and 7 during sexual induction before isolation (a) and analysis of AMA1 expression in sporozoites by immunofluorescence analysis (b). AMA1 expression in sporozoites was detected using anti-HA, while anti-HSP70 was used to detect sporozoites. Anti-GFP antibodies were used to identify successful recombination upon rapamycin (RAP) treatment. The results show the absence of HA expression in 75% of rapamycin-treated sporozoites compared with 100% HA expression in DMSO-treated parasites, confirming AMA1 excision. Unexpectedly, GFP expression is detected in sporozoites irrespective of treatment conditions, indicating transcription of the promoterless GFP cassette in sporozoites, but not in asexual stages. Download FIG S5, TIF file, 0.5 MB. Copyright © 2019 Tibúrcio et al. 2019 Tibúrcio et al. This content is distributed under the terms of the Creative Commons Attribution 4.0 International license.

### FIKK7.1 conditional KO characterization across the P. falciparum life cycle.

**FIG 3:**
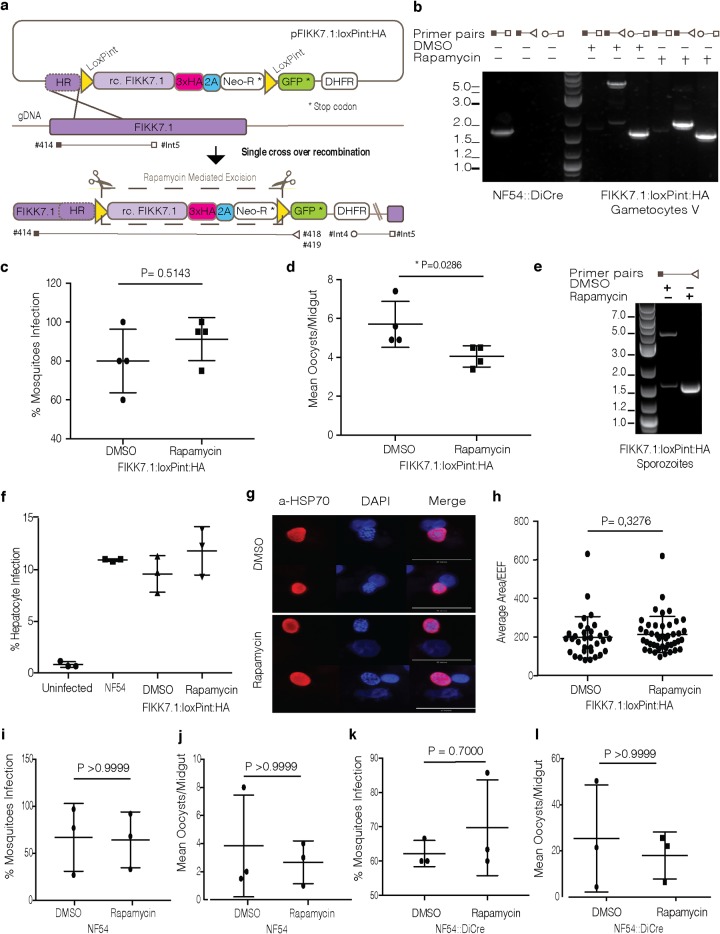
Generation and characterization of a FIKK7.1:loxPint:HA conditional KO across the P. falciparum life cycle. (a) Description of the strategy used to make an FIKK7.1 conditional KO line (FIKK7.1:loxPint:HA). The rescue plasmid contains a recodonized version of FIKK7.1 kinase domain followed by a triple-HA, T2A peptide, and neomycin resistance cassette, flanked by loxPints. A GFP cassette is inserted to facilitate efficient excision events. The primer pairs represented here were used to test correct integration of FIKK7.1:loxPint:HA, and efficient rapamycin-mediated excision and can be found in Table S1. (b) PCR analysis shows correct integration of FIKK7.1:loxPint:HA and near complete excision of FIKK7.1 kinase domain after rapamycin treatment. (c) Infection rates in A. stephensi fed DMSO- or rapamycin-treated FIKK7.1:loxPint:HA gametocytes. Each data point represents the value for an independent experiment. (d) Mean number of oocysts per mosquito gut resulting from DMSO- or rapamycin-treated FIKK7.1:loxPint:HA parasites where each data point represents the value from an independent experiment. (e) PCR analysis of sporozoites isolated from mosquitoes infected with DMSO- or rapamycin-treated gametocytes. (f) Hepatocyte infection rates comparing uninfected, NF54, and DMSO- and rapamycin-treated FIKK7.1:loxPint:HA parasites. The data points represent the values for technical replicates of one experiment. (g) Immunofluorescence analysis of sporozoite development in human hepatocytes. Anti-HSP70 antibodies were used to identify and compare the EEFs at day 5 postinvasion. (h) Measurement of the area of EEFs on day 5 postinvasion, where each EEF from two independent experiments is represented. (i) Infection rates in A. stephensi mosquitoes fed DMSO- or rapamycin-treated NF54 gametocytes. Each data point represents the value from an independent experiment. (j) Mean number of oocysts per mosquito gut resulting from DMSO- or rapamycin-treated NF54 parasites where each data point represents the value for an independent experiment. (k) Infection rates in A. gambiae fed DMSO- or rapamycin-treated NF54::DiCre gametocytes. Each data point represents the value from an independent experiment. (l) Mean number of oocysts per A. gambiae mosquito gut resulting from DMSO- or rapamycin-treated NF54::DiCre parasites. Each data point represents the value from an independent experiment. All *P* values were calculated by the Mann-Whitney *t* test.

Because of the unexpected phenotype of AMA1 during transmission, we wanted to confirm that rapamycin treatment itself has no effect on transmission. To do so, we generated a conditional KO line of FIKK7.1 (PF3D7_0726200), a nonessential blood-stage kinase, in the NF54::DiCre line (25). We flanked the *fikk7.1* kinase domain with loxPints (13) and used a selection-linked integration (SLI) method to select for integration (10) (Fig. 3a). To understand whether gene deletion during sexual development was affecting mosquito infection, we conditionally deleted *fikk7.1* during sexual development (Fig. 3b). The rapamycin-treated gametocytes were grown to maturity and then fed to A. stephensi to evaluate transmission efficiency. Rapamycin treatment of the FIKK7.1:loxPint:HA gametocytes showed efficient excision of the floxed locus, as expected (Fig. 3b). DMSO- and rapamycin-treated parasites showed no differences in the number of infected mosquitoes (Fig. 3c), although rapamycin treatment resulted in a modest but significant reduction (21%) of oocyst loads (Fig. 3d). Sporozoites from both conditions were then extracted from the mosquito salivary glands and used for PCR analysis and invasion assays (Fig. 3e and f). Genomic DNA analysis showed some background excision in the DMSO condition, while highly efficient excision of the *fikk7.1* kinase domain was detected in rapamycin-treated cultures (Fig. 3e). Isolated sporozoites showed normal hepatocyte invasion rates (Fig. 3f) and development of EEFs (Fig. 3g and h) when comparing NF54 and FIKK7.1:loxPint:HA DMSO- and rapamycin-treated parasites.

To better understand whether the observed reduction in oocyst numbers was due to rapamycin treatment or indeed FIKK7.1 specific, we treated NF54 gametocytes with DMSO or rapamycin and compared parasite infectivity to mosquito infectivity and number of oocysts. This analysis showed no difference in mosquito infection rates; however, a 30% reduction of oocysts in rapamycin-treated parasites compared to DMSO-treated parasites was observed (Fig. 3i and j). Although the variation in the oocyst numbers was high and the differences were not statistically significant, we observed a trend, which is discussed further below.

These results showed that efficient rapamycin-mediated gene deletion can be achieved in sexual stages without substantially impacting later stages of parasite development. Furthermore, we confirmed a nonessential role of FIKK7.1 throughout the P. falciparum life cycle and that genetically modified and rapamycin-treated parasites can be transmitted by mosquitoes and infect hepatocytes.

### Anopheles gambiae *TEP1* knockdown can be infected with NF54::DiCre.

It has been shown previously that Pfs47-KO parasites fail to infect A. gambiae mosquitoes because of efficient ookinete killing by the mosquito complement-like protein TEP1 (26, 27). We reasoned that depletion of the TEP1 protein in A. gambiae would also expand the usability of the NF54::DiCre strain in this mosquito species. To test the infectivity of NF54::DiCre in A. gambiae, we used a transgenic immunodeficient mosquito line depleted for *TEP1* (28). NF54::DiCre parasites efficiently infected A. gambiae 7b mosquitoes and produced similar oocyst numbers to the parental NF54 parasites (Fig. 3k and l). We concluded that NF54::DiCre line can be used to infect A. gambiae mosquitoes. Similar to A. stephensi infections, rapamycin-treated parasites did not show any difference in prevalence of infection (Fig. 3k), but they displayed lower oocyst numbers (30% lower) compared to DMSO controls (Fig. 3l). However, the observed differences were not statistically significant, probably due to high variability between mosquito infection experiments (Fig. 3l). These results show that the NF54::DiCre line can be used for infection of A. gambiae species in complement-depleted mosquitoes. However, the observed effect of rapamycin treatment on oocyst loads calls for further investigation.

## DISCUSSION

The availability of genetic tools to manipulate P. falciparum is fundamental in the study of malaria and identification of novel drug targets. By introducing a DiCre cassette into the *pfs47* locus of NF54 parasites using CRISPR/Cas9, we created a marker-free NF54::DiCre parasite line that develops through the P. falciparum life cycle up to the stage of hepatocyte development. Our study introduces a novel tool and methodology that allows for the first time the conditional deletion of essential genes during sexual and liver stages in human malaria parasites. To validate the use of this tool, we made an *AMA1* conditional KO line to truncate the gene at different developmental stages. AMA1 is a microneme protein that plays a critical role during erythrocyte invasion, and due to its essential role during asexual growth, it cannot be genetically disrupted by conventional methods. AMA1 is also abundantly expressed in sporozoites; however, the role of AMA1 during hepatocyte invasion is contradictory. While stage-specific promoter-driven conditional disruption of AMA1 in the rodent malaria parasite P. berghei had no effect on cell traversal activity or hepatocyte invasion (22), invasion of P. falciparum sporozoites was drastically reduced upon incubation with the anti-AMA1 monoclonal antibodies or the AMA1 inhibitory peptide R1 (23, 24). Using a conditional KO, we aimed to address the role of AMA1 during hepatocyte invasion. However, an unexpected significant reduction of oocyst (82%) and sporozoite numbers in rapamycin-treated parasites made these experiments impossible. The basis for this reduction is currently unknown. No AMA1 has been identified in the P. berghei ookinete proteome (29, 30), and no P. falciparum ookinete proteome data are available as a comparison. In line with micronemes and microneme-produced proteins being present in all invasive zoite forms, including ookinetes, transcriptomic data revealed an upregulation of AMA1 in ookinetes ( 31 ). In light of our results, it is possible that AMA1 regulates P. falciparum ookinete invasion in mosquitoes. However, further work is required to explore a putative role of AMA1 during mosquito transmission.

To examine the effect of rapamycin treatment on the transmissible forms of P. falciparum, we generated a FIKK7.1 conditional KO line. We predicted that FIKK7.1 is not required for parasite development in mosquitoes. However, rapamycin treatment of the parasites during sexual development *in vitro* reduced the mean oocyst numbers in the midguts of *A stephensi* mosquitoes. While the observed differences for FIKK7.1 were statistically significant, a similar mean decrease in oocyst numbers were observed in rapamycin-treated control NF54 P. falciparum parasites in A. stephensi and A. gambiae infections, providing a possible explanation for the FIKK7.1 KO phenotype. However, the FIKK7.1 KO data differed from the *AMA1* KO data, which showed a significant fourfold reduction in oocyst numbers, indicating that rapamycin treatment *per se* is not responsible for the AMA1 phenotype within mosquito parasite development. Moreover, rapamycin treatment did not affect the percentage of infected mosquitoes, whereas a strong significant reduction was observed in infection prevalence for *AMA1* KO line. Rapamycin treatment did not affect either the hepatocyte infection rate or the size of extraerythrocytic forms, indicating that it does not curb the transmission of the NF54::DiCre parasites in general.

In summary, we show that the NF54::DiCre parasite lines can be used to generate conditional KOs of essential and nonessential genes in different developmental stages and assess their role throughout the P. falciparum life cycle. We have not tested whether deletion of genes can be achieved during the mosquito phase, for example by feeding mosquitoes with rapamycin. Such experiments will require very careful analysis because of potential toxic effects of the mTOR (mammalian target of rapamycin) inhibitor rapamycin on mosquito physiology. However, the ability to delete genes in gametocytes and hepatocytes should generate unprecedented insights into the biology and drug target validation across parasite stages. The ability to delete blood-stage essential genes, such as *ama1*, in sexual or liver-stage parasites may also enable the generation of late-stage arrested attenuated parasites for clinical vaccine development. We believe that this tool will promote substantial advancements in research of transmission and liver stages and will therefore open up new avenues for the study of one of the world’s deadliest diseases.

## MATERIALS AND METHODS

### Plasmodium falciparum *in vitro* culture of asexual and sexual blood stages.

The Plasmodium falciparum parasite lines used in this study were all derived from the NF54 strain (originally isolated from an imported malaria case in the Netherlands in the 1980s) (BEI Resources catalog no. MRA-1000) (32). Asexual parasites were cultured in human blood (UK National Blood Transfusion Service) and RPMI 1640 medium containing 0.5% (wt/vol) AlbumaxII (Invitrogen) at 37°C as previously described (33). Asexual parasites were used to produce gametocytes by seeding asexual rings at 1% parasitemia and 4% hematocrit on day 0 and feeding the parasites once a day during 15 days (day 0 to day 14) in 3% O_2_–5% CO_2_–92% N_2_ gas in RPMI 1640 medium complemented with 25 mM HEPES, 50 mg/liter hypoxanthine, 2 g/liter sodium bicarbonate, and 10% human serum (34).

### Conditional knockout (KO) induction at different P. falciparum developmental stages.

To induce DiCre-driven loxP site recombination, synchronized ring-stage parasites were treated with 100 nM rapamycin (Sigma) or dimethyl sulfoxide (DMSO) (0.1% [vol/vol]) for 18 h. Parasites were subsequently washed twice with RPMI 1640 medium and returned to culture. Schizonts used for PCR, immunofluorescence assay (IFA), or protein extraction were taken 38 to 41 h after rapamycin or DMSO treatment. The rapamycin-induced recombination in early sexual stages was accomplished by treating the parasites with 100 nM rapamycin or DMSO for 4 h on day 6 and on day 7 during sexual induction. The parasites were subsequently washed in RPMI 1640 medium and used for further analysis or mosquito feeding on day 14. During liver development, the exoerythrocytic forms (EEFs) were treated with 100 nM rapamycin 72 h after invasion for 24 h and sequentially washed with culture medium.

### Exflagellation assay.

As previously described, sexual induction starts on day 0 by seeding asexual rings at 1% parasitemia (34). On day 14, male exflagellation was induced by adding 5 μl of ookinete medium (RPMI 1640 supplemented with 25 mM HEPES, 50 μg ml^−1^ hypoxanthine, 2 g liter^−1^ NaHCO_3_, and 100 μM xanthurenic acid) to a 100-μl sample of gametocyte culture in a 96-well plate (34). Twenty minutes after gamete activation, exflagellation was recorded by bright-field microscopy using a Nikon Eclipse Ti wide-field inverted microscope using a 4× objective, 1.5× zoom, recording a 10-frame video time-lapse over 2 s for each well, as previously described (32). Two independent experiments, each with at least three technical replicates, were used per condition. Exflagellation was quantified using the open-source ICY Image Analysis software (35). The Mann-Whitney *t* test was used for calculating statistical significance (*P* < 0.05).

### Standard membrane feeding assay.

For the standard membrane feeding assay (SMFA) experiments in Anopheles stephensi, sexual development was induced by seeding asexual rings at 1% parasitemia and 5% hematocrit (O red blood cells from healthy Dutch blood bank donors without any history of malaria) in RPMI 1640 with HEPES (5.94 g/liter), hypoxanthine (0.05 g/liter]), 10% human serum, and 5% bicarbonate (42 ml/liter) and cultured in an automated tipper system (36, 37) and changing the medium twice a day for 14 days. On day 14, the cultures containing mature gametocytes were used for the SMFA as previously described (38). For SMFA on Anopheles gambiae 7b mosquitoes (28), parasites were grown in culture flasks on O^+^ human red blood cells (Haema, Berlin, Germany). Sexual development was induced by seeding parasites at 4% parasitemia and daily medium changes. Rapamycin (100 nM) or DMSO (0.1%) treatments were applied for 24 h on day 3 after seeding, corresponding to I-II stage gametocytes *in vitro*. Infections were done on day 12 after seeding. Mosquitoes were fed for 15 min on a membrane feeder with the gametocytes and kept in a secured S3 laboratory according to the national regulations (Landesamt für Gesundheit und Soziales, project 411/08). Unfed mosquitoes were removed after feeding, and fed mosquitoes were maintained at 26°C until dissected for oocyst count.

### Isolation and purification of P. falciparum sporozoites.

Fourteen to 21 days postinfection of female A. stephensi mosquitoes, the salivary glands were hand dissected, collected in complete William’s B medium (see “Isolation and culture of human hepatocytes” below), and homogenized in a homemade glass grinder. Sporozoites were counted in a Burker-Turk counting chamber using phase-contrast microscopy (39).

### Isolation and culture of human hepatocytes.

Primary human hepatocytes were prepared from liver segments taken from adult patients during liver surgery, in agreement with Dutch ethical regulations. Hepatocytes were isolated by using the two-step enzymatic perfusion technique (40). The liver tissue was perfused via any vessel (venous or portal vein) with 500 ml of HBSS medium (Gibco catalog no. 14170-088) supplemented with 10 mM HEPES (Gibco catalog no. 15630-056) and 0.64 mM EDTA (Invitrogen catalog no. 15575-038). Subsequent perfusion was performed with 500 ml of HBSS medium supplemented with 10 mM HEPES and with 100 ml of oxygenized HBSS medium supplemented with 10 mM HEPES, 0.75 mg/ml CaCl_2_, and low concentrations of collagenase (3,333 U per 50 ml). Next, the tissue was perfused with 100 ml of oxygenized HBSS medium supplemented with 10 mM HEPES, 0.75 mg/ml CaCl_2_, and high concentrations of collagenase (13,333 U per 50 ml). This buffer was recirculated until liver tissue became very soft. Subsequently, the liver tissue was transferred into a petri dish containing 40 ml cold DMEM medium (Gibco catalog no. 31885-023) supplemented with 10% fetal bovine serum (FBS) (Gibco catalog no. 10270). The liver tissue was cut into small pieces to extract cells for a single-cell suspension of hepatocytes. This primary human hepatocyte suspension was centrifuged at 10 × *g* with low brake for 5 min at 4°C. Hepatocyte pellets were washed in Dulbecco modified Eagle medium (DMEM) without serum and again centrifuged at 50 × *g* with low brake for 5 min at room temperature. This step was repeated until the supernatant looked clear. Viable cells were separated from dead cells using Percoll purification (final concentration of 28.8% [vol/vol]), and the pellet was resuspended in complete William’s B medium, consisting of William’s E medium with Glutamax (Gibco catalog no. 32551-087) supplemented with 10% heat-inactivated human serum, 1% insulin-transferrin-selenium (Gibco catalog no. 41400-045), 1% sodium pyruvate (Gibco catalog no. 11360-036), 1% minimum essential medium nonessential amino acids (MEM-NEAA) (Gibco catalog no. 1140-035), 1% amphotericin B (Fungizone) antimycotic (Gibco catalog no. 15290-018), 2% penicillin-streptomycin (Gibco catalog no. 15140-122), and 1.6 μM dexamethasone. Hepatocytes (260,000 cells/coverslip) were plated on collagen-coated 12-cm wells at 37°C in an atmosphere of 5% CO_2_, and the medium was refreshed the next morning (500 μl/well complete William’s B medium) and then every 2 days.

### Standard sporozoite infectivity assay.

Fresh human hepatocytes were seeded in 24-well culture plates (250,000 cells/well) and incubated at 37°C for 48 h before inoculation with P. falciparum sporozoites. Sporozoites in culture medium were added to the wells and coincubated with the confluent human hepatocytes (1 sporozoite:1 hepatocyte) for 3 h at 37°C (for sporozoite invasion). After 3 h, the wells were washed to remove sporozoites that have not invaded hepatocytes and then incubated at 37°C with daily medium refreshment for 7 days to obtain liver schizonts.

### Nested PCR.

The sporozoite’s genomic DNA (gDNA) for genotype analysis was extracted using a Qiagen DNeasy blood and tissue kit. The first PCR was done using primers F0 and R1 (see Table S1 in the supplemental material) and OneTaq 2× Master Mix (NEB). Five microliters of the first PCR were then used as a template for a second pair of primers, primers #420 and #423 (Table S1), and a second amplification step using OneTaq 2× Master Mix (NEB).

### Western blotting.

Schizonts were released from erythrocytes by the addition of phosphate-buffered saline (PBS) containing 0.15% (wt/vol) saponin and protease inhibitors (cOmplete EDTA-free; Sigma) for 3 min on ice. Saponin lysates were solubilized with 3× sample buffer with 5% beta-mercaptoethanol at a concentration of 1.6 × 10^9^ parasites/ml. Parasite extracts were subjected to SDS-PAGE and transferred to a nitrocellulose membrane. Membranes were immunostained with rat antihemagglutinin (anti-HA) (1:1,000 dilution; Roche) and rabbit anti-P. falciparum EBA175 (anti-PfEBA175) (1:2,000 dilution; a kind gift from Christine R. Collins), followed by IRDye 680LT goat anti-rat IgG (1:10,000 dilution; LI-COR) and IRDye 800CW goat anti-rabbit IgG (1:10,000 dilution; LI-COR). The signals were detected by a fluorescence imager (Odyssey CLx; LI-COR).

### Immunofluorescence assay at different parasite stages.

Air-dried blood films of asexual parasites were fixed with 4% paraformaldehyde containing 0.0075% glutaraldehyde for 15 min and permeabilized in 0.1% (vol/vol) Triton X-100 (Sigma) for 10 min. Blocking was performed in 3% bovine serum albumin (BSA) for 1 h. Slides were incubated with rat anti-HA (1:1,000 dilution; Roche) at room temperature for 30 min, followed by Alexa Fluor-conjugated goat anti-rat IgG (1:1,000 dilution) at room temperature for 30 min. Parasite nuclei were stained with 4′,6′-diamidino-2-phenylindole (DAPI) (Invitrogen). Slides were mounted with ProLong Gold antifade reagent (Invitrogen), and images were obtained with the inverted fluorescence microscope (Ti-E; Nikon, Japan) and processed using NIS-Elements software (Nikon, Japan).

Sporozoites were extracted from the salivary glands of A. stephensi mosquitoes, fixed in 4% paraformaldehyde, permeabilized with 1% Triton X-100, and stained using rabbit anti-HSP70 (StressMarq SPC-186 used at 1:75 dilution), and rat anti-HA (Sigma-Aldrich catalog no. 11867423001) (used at 1:500 for 1 h at room temperature). All secondary antibodies were used at 1:200 dilution and incubated for 1 h at room temperature: goat anti-rat secondary antibody conjugated to Alexa Fluor 594 (goat anti-rat Alexa Fluor 594) (ThermoFisher catalog no. A-11007) and donkey anti-rabbit Alexa Fluor 647 (ThermoFisher catalog no. A-31573). The staining is completed with DAPI staining (ThermoFisher catalog no. D1306) and rabbit anti-green fluorescent protein (anti-GFP) (ThermoFisher catalog no. A-21311; used at 1:200) for 1 h in 1× PBS and mounted using Vectashield (Vector Laboratories catalog no. H-1000). Infected hepatocytes were fixed on day 5 or 7 postinvasion and permeabilized as mentioned above. The EEFs were then stained using rabbit anti-HSP70 (StressMarq SPC-186 used at 1:75 dilution), rat anti-HA (Sigma-Aldrich catalog no. 11867423001; used at 1:500), and mouse anti-MSP1 (Sanaria AD233; 1:100) for 1 h at room temperature. All secondary antibodies were used at 1:200 dilution and incubated for 1 h at room temperature: donkey anti-mouse Alexa Fluor 694 (ThermoFisher catalog no. A-31571), goat anti-rat Alexa Fluor 594 (ThermoFisher catalog no. A-11007), and chicken anti-rabbit Alexa Fluor 488 (ThermoFisher catalog no. A-21441). All secondary antibodies were used at 1:200 dilution and incubated for 1 h at room temperature: goat anti-rat Alexa Fluor 594 (ThermoFisher catalog no. A-11007) and donkey anti-rabbit Alexa Fluor 647 (ThermoFisher catalog no. A-31573). The staining is completed with DAPI staining (ThermoFisher catalog no. D1306) for 1 h in 1× PBS and mounted using Vectashield (Vector Laboratories catalog no. H-1000). For both sporozoite and hepatic stages, the coverslips are tile scanned (9 × 9) using the Leica DM16000B automated high-content microscope with a 20× objective. The resulting number of EEFs and DAPI-stained cells were counted; the infection rate is the percentage of hepatocytes that has an intracellular parasite. High-resolution microscopy was performed with the Zeiss LSM880 with Airyscan at 63× oil objective.

10.1128/mBio.01170-19.6 TEXT S1 Supplemental Materials and Methods not contained in the main article, including Plasmid construction and transfection, DNA preparation and analysis for whole-genome sequencing and macrogamete assays, including relevant references. Download Text S1, DOCX file, 0.04 MB. Copyright © 2019 Tibúrcio et al. 2019 Tibúrcio et al. This content is distributed under the terms of the Creative Commons Attribution 4.0 International license.

10.1128/mBio.01170-19.8 TABLE S2 Primers used to generate FIKK7.1:loxPint:HA and Ama1:loxPint:HA plasmids. Download Table S2, DOCX file, 0.02 MB. Copyright © 2019 Tibúrcio et al. 2019 Tibúrcio et al. This content is distributed under the terms of the Creative Commons Attribution 4.0 International license.
