# Supplementary figures and images for: A Novel Tool for the Generation of Conditional Knockouts To Study Gene Function across the Plasmodium falciparum Life Cycle
Source: mBio. 2019 Sep 17;10(5):e01170-19. doi: 10.1128/mBio.01170-19 (PMC6751054; doi:10.1128/mBio.01170-19)

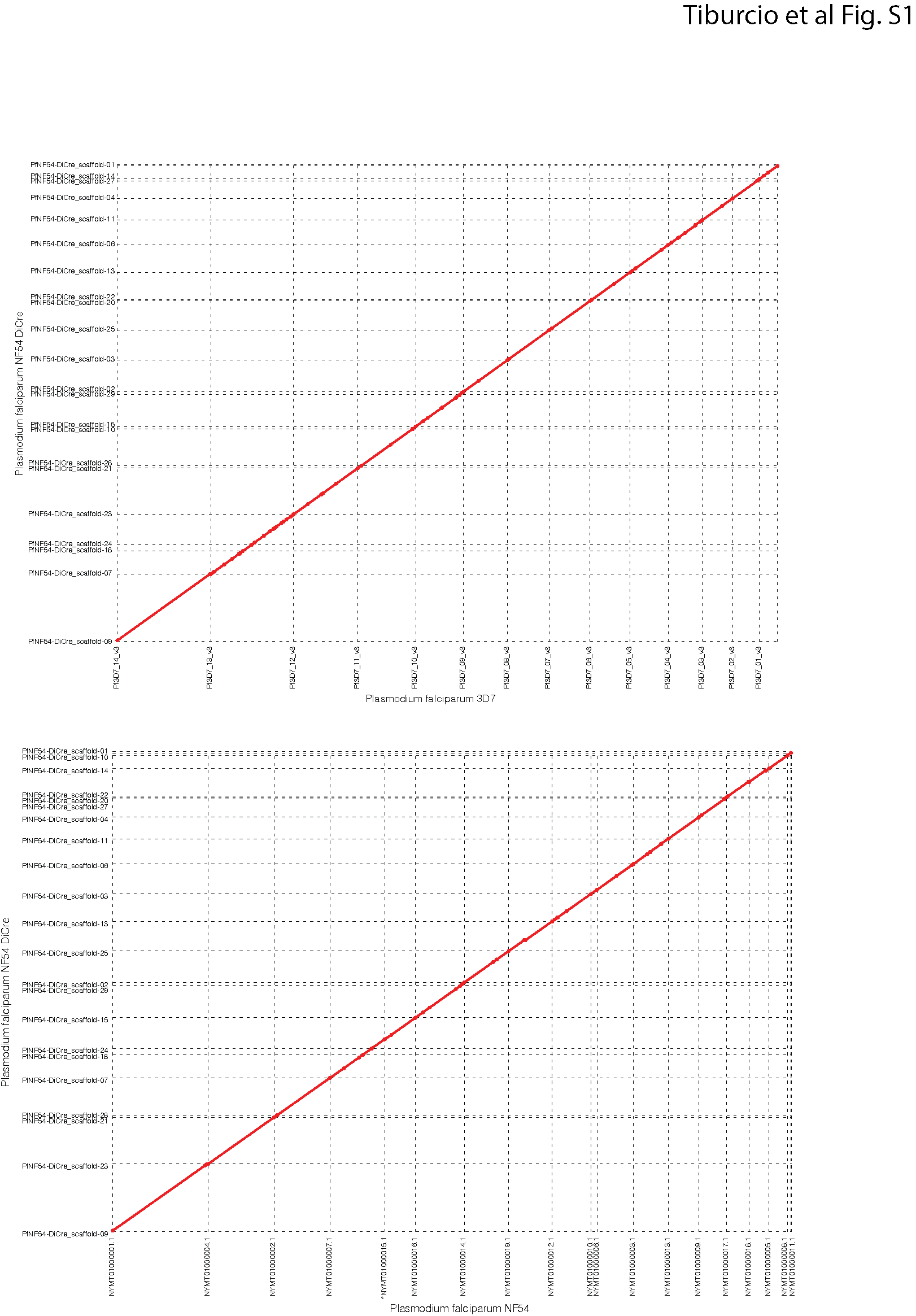

Supplement: FIG S1 [file mBio.01170-19-sf001.tif]

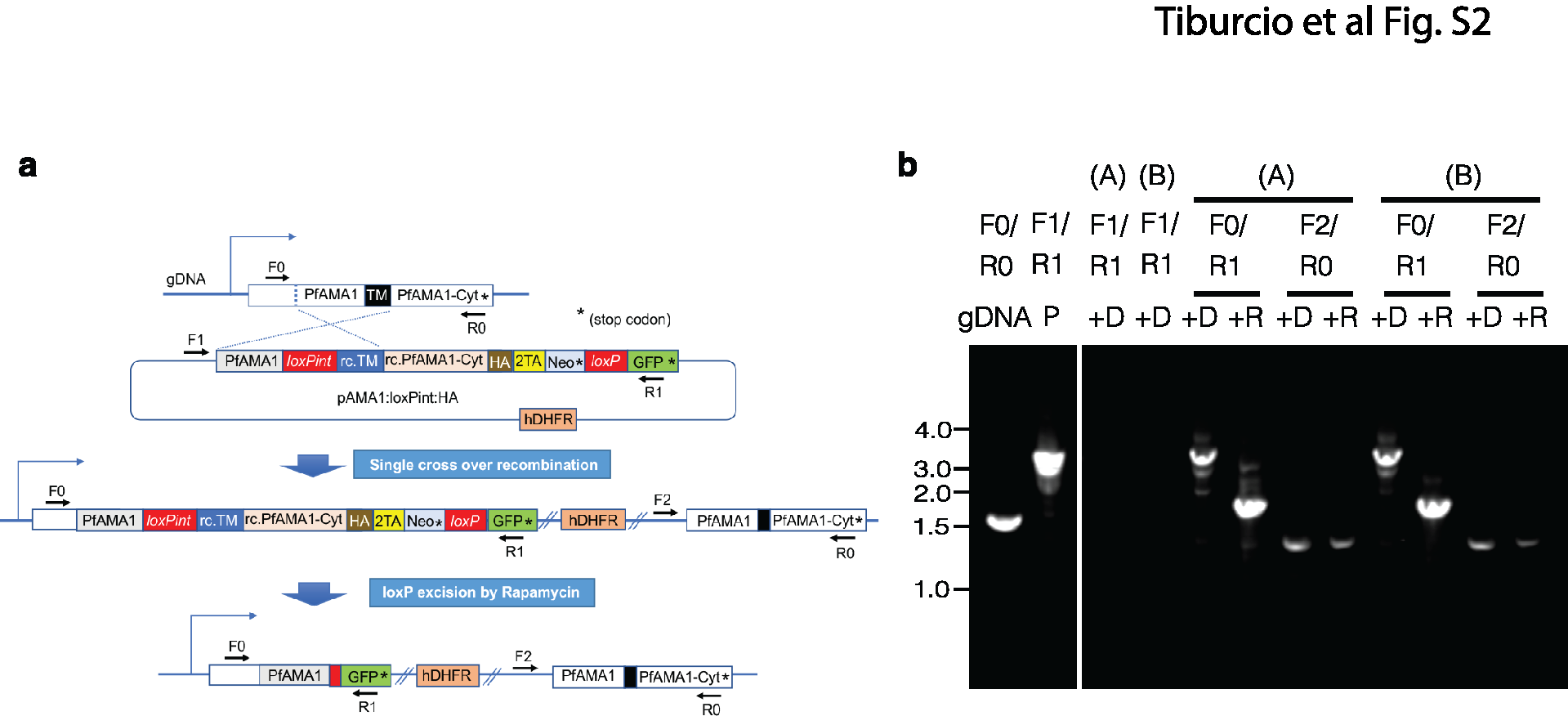

Supplement: FIG S2 [file mBio.01170-19-sf002.tif]

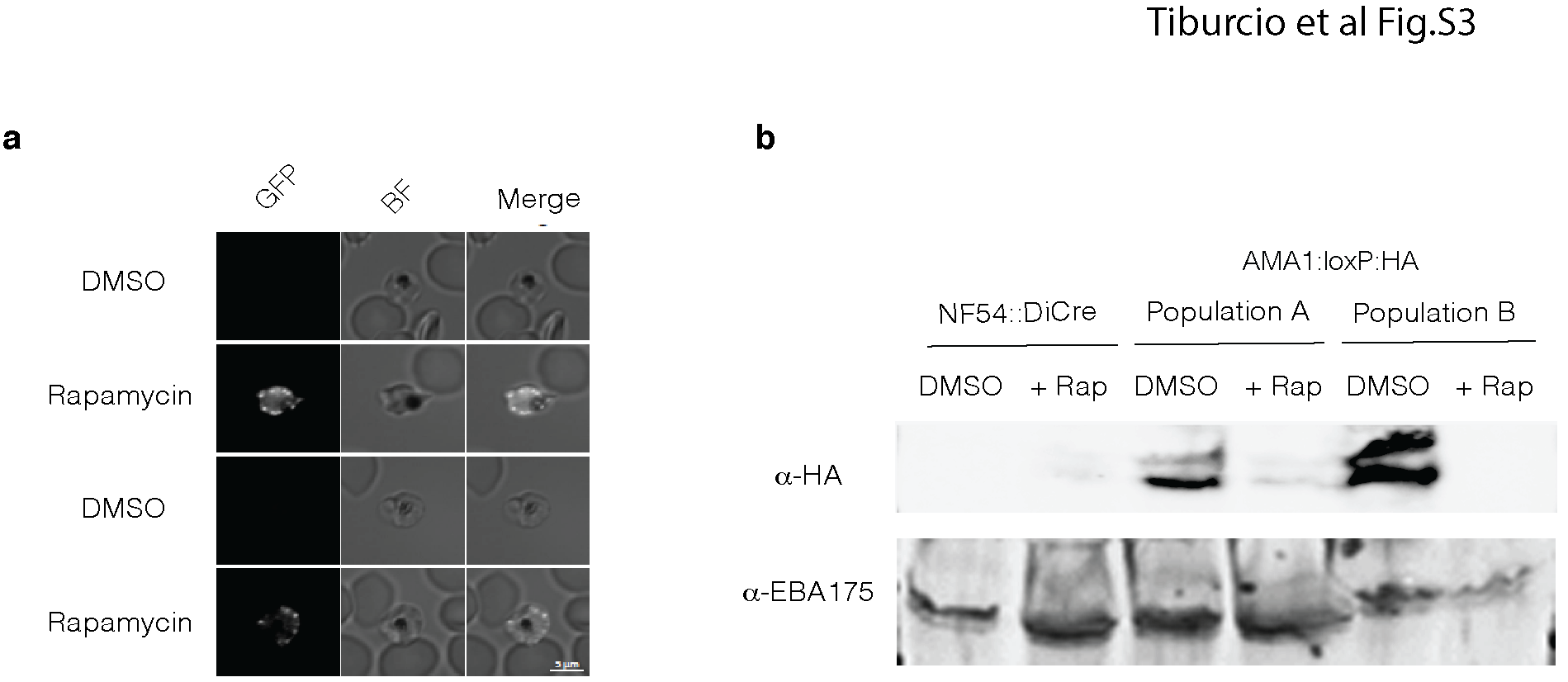

Supplement: FIG S3 [file mBio.01170-19-sf003.tif]

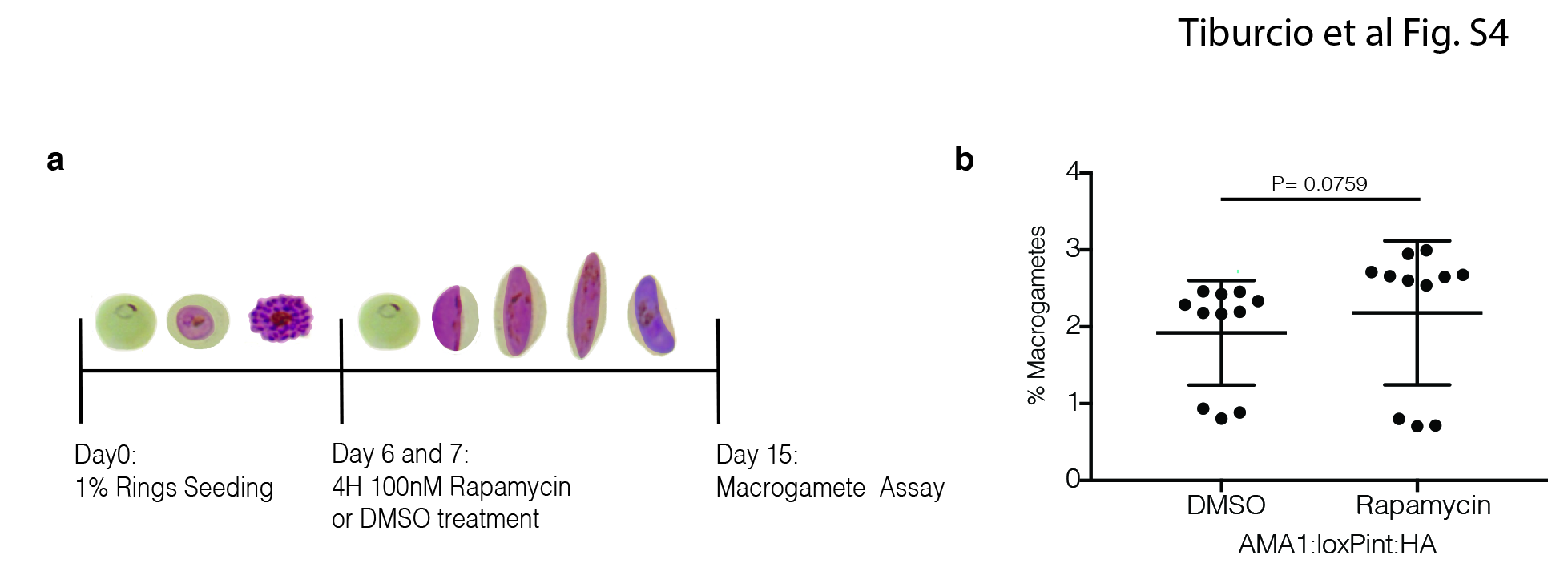

Supplement: FIG S4 [file mBio.01170-19-sf004.tif]

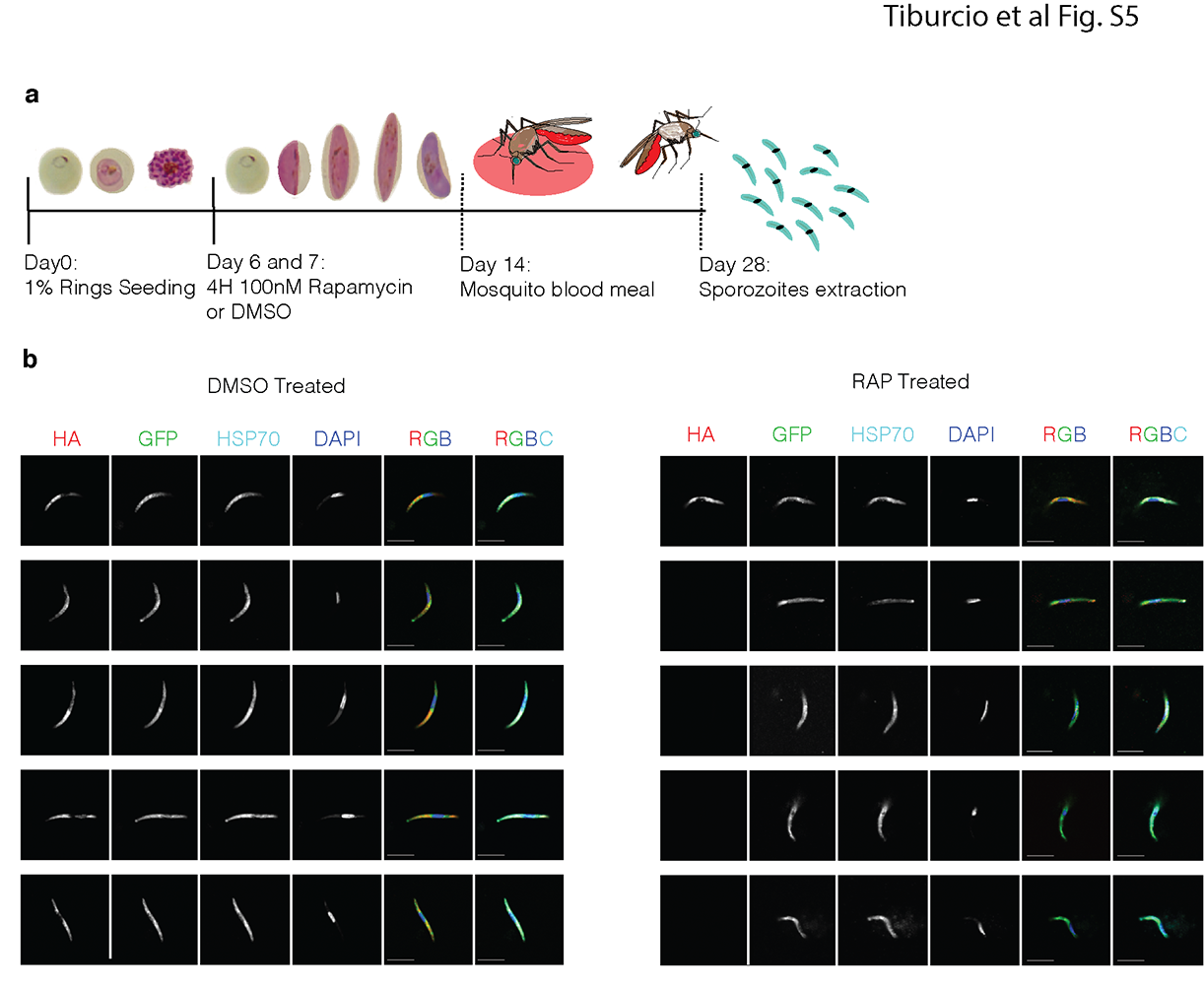

Supplement: FIG S5 [file mBio.01170-19-sf005.tif]
