# Supplementary material for: A Novel Tool for the Generation of Conditional Knockouts To Study Gene Function across the Plasmodium falciparum Life Cycle
Source: mBio. 2019 Sep 17;10(5):e01170-19. doi: 10.1128/mBio.01170-19 (PMC6751054; doi:10.1128/mBio.01170-19)
Supplement: TABLE S1 [file mBio.01170-19-st001.docx]

**Table S1**

| Primer | Sequence |
| --- | --- |
| #128 | ATGCTCCTGTCTGTGTGCAGATCC |
| #135 | AAATTGCCAGAATCAGAGTGAAGG |
| #151 | ATTGCATACACATAAATATTTGTGTTGTAC |
| #152 | CACATACGTATTGTGTTGAGCTTAATAG |
| #154 | GATGCGATATGTAATTCCATTACTGC |
| #153 | CTAATGTTAAGCCAACTGTAGTTGGG |
| #414 | ATTCACTCTATATTTCCTAAAAAGTCC |
| #418 | ATGGAACTGGTAGTTTTCCAGTAGTGC |
| #419 | AGTATTTTGTTGATAATGGTCTGC |
| #420 | AGATATACCACATGTAAATGAATTTCC |
| #423 | ATGGAACTGGTAGTTTTCCAGTAGTGC |
| Int4 | CCCCAGGCTTTACACTTTATGCTTCCGGCTC |
| Int5 | CCACCACGGATGAATGCCT |
| AMA1-F0 | CGAACCCGCACCACAAGAAC |
| AMA1-R0 | ACTGGTGTTGTATGTGATGCTC |
| AMA1-F1 | TGATTACGCCAAGCTATTTAGG |
| AMA1-R1 | CTTTTGTTTGTCTGCCATGATG |
