# Supplementary material for: A Novel Tool for the Generation of Conditional Knockouts To Study Gene Function across the Plasmodium falciparum Life Cycle
Source: mBio. 2019 Sep 17;10(5):e01170-19. doi: 10.1128/mBio.01170-19 (PMC6751054; doi:10.1128/mBio.01170-19)
Supplement: TEXT S1 [file mBio.01170-19-s0001.docx]

**Supplemental Material and Methods**

**Plasmid construction and transfection.** The generation of the DiCre marker free transgenic line was accomplished by transfecting the *P. falciparum* NF54 line with 60 μg of linearized rescue plasmid pBSPfs47DiCre containing the DiCre cassette and 20–30 μg of CRISPR/Cas9 plasmid pDC287 containing the guide RNA targeting the Pfs47 locus, as previously described (1). The resulting DiCre expressing line was named NF54::DiCre and has been used to produce conditional KO lines described below. To generate the conditional gene knockout plasmid for *P. falciparum* AMA1 (PF3D7_1133400), the targeting fragment of *ama1* was amplified by PCR from *P. falciparum* NF54 genomic DNA using primer set BglII-AMA1-F/AMA1-R2. The internal loxP fragment was amplified from pFIKK7.1:loxPint:HA using the primer set AMA1-ILI-F2/AMA1rec-ILI-R2. Recodonised AMA1 containing a C-terminal HA tag (RecAMA1-HA-SalI) was synthesized by Geneart and amplified by PCR with primers synAMA1-F2/HD126. Three fragments were joined together with the backbone fragment of BglII and SalI digested pFIKK7.1:loxPint:HA using Gibson assembly (NEB) resulting in the plasmid pAMA1:loxPint:HA. The FIKK7.1 (PF3D7_0726200) conditional KO was designed based on the pARL1a(+) plasmid containing a GFP cassette and a DHFR inhibitor resistance cassette (a kind gift of Tobias Spielmann, Bernhard Nocht Institute for Tropical Medicine, Hamburg, Germany). The FIKK7.1 plasmid used to make the conditional KO consists of a 5’ FIKK7.1 homology region and the recodonized FIKK7.1 kinase domain flanked by loxPint on 5’ end and a triple HA tag on 3’ end followed by a T2a-Neomycin cassette flanked by a second loxPint on the 3’ end in the pARL1a(+) plasmid backbone containing a GFP cassette and a DHFR inhibitor resistance cassette. The targeting fragment of FIKK7.1 was amplified by PCR from *P. falciparum* 1G5 genomic DNA using primer set FIKK7.1HRForward/FIKK7.1HRReverse (Supplementary Table 2). The recodonized version of FIKK7.1 kinase domain fused to a triple HA tag on the 3' end and flanked with 2 LoxP introns was ordered from Geneart. The T2a-Neomycin cassette was amplified with primer set T2a-NeoForward/T2a-NeoReverse (Supplementary Table 1) and Gibson assembled in the Geneart ordered plasmid after AvaI restriction digest and named PFIKK7.1:loxPint:HA:T2a-Neo. Finally, FIKK7.1 homology region fragment and PFIKK7.1:loxPint:HA:T2a-Neo PCR amplified fragment using FIKK7.1RCForward/FIKKUniversalReverse primer pair (Supplementary Table 2) were Gibson assembled in the BglII and NcoI digested pARL1a(+) plasmid backbone resulting in the plasmid pFIKK7.1:loxPint:HA. All plasmids were suspended in 100uL of P3 primary cell solution containing 40 to 70ug DNA. Transfection was performed using Amaxa 4D-Nucleofector^TM^ (Lonza). Briefly, purified P. falciparum NF54::DiCre schizont stages were electroporated using program FP158 (2). Selection of parasites transfected with AMA1 and FIKK7.1 was done using 5nM WR99210 (Jacobus Pharmaceutical) and after a first round of selection, a double selection with 225 μg/mL G418 was used (3). WR99210 and neomycin resistant FIKK7.1:loxPint:HA conditional KO parasites were further selected by limiting dilution cloning in microplates at a 0.5 parasite per well (4). Parasite genomic DNA (gDNA) for genotype analysis was extracted using a Qiagen DNeasy Bood. All primers used in the construction and integration of the constructs as well as confirmation of rapamycin mediated excision are described in Supplementary Table 1 and 2.

**DNA extraction and sequencing.** Late stage parasites were grown in six mL of concentrated blood at 3.5% parasitemia and enriched by plasmagel flotation. DNA was extracted with the Qiagen Genomic Tip 500/G kit (Qiagen #10262) and further purified with phenol/chloroform. Library preparation and sequencing were performed by GATC Biotech (Konstanz, Germany) on a PacBio RS II sequencer with an average read length of 13,877 base pairs and genome coverage of ~150 x post-filtering. The same genomic DNA was sonicated and libraries were prepared with the NEBNext Ultra II DNA Library preparation kit (New England Biolabs, #E7645S) and sequenced on the Illumina NextSeq 500 sequencing platform to an average genome coverage of ~785x.

**Genome assembly.** The genome was assembled using the PacBio RS_Assembly_HGAP.3 protocol (included in the SMART Portal v2.3.0) with default settings and a target genome size of 23 megabases (5). The initial assembly was scaffolded using SSPACE-LongRead (6) and super-scaffolds were generated using pyscaf (https://github.com/lpryszcz/pyScaf) and the 3D7 genome as reference. Resulting gaps were closed with GapFiller (7) using the Illumina short reads. The assembly was further polished with the PacBio reads using ‘blasr’ (8) and ‘quiver’ {Chin, 2013 #53;Chaisson, 2012 #56}. Illumina short reads were subsequently mapped to the assembly using bowtie2 (9), and read alignments were used to fix remaining SNPs, short indels, and breaks using ‘pilon’ (10). The apicoplast and mitochondrial genomes were assembled from raw PacBio reads using Organelle_PBA (11) using the 3D7 sequences as reference.

**Genome annotation and accession number(s).** A liftover annotation was performed using ‘flo’ (https://github.com/wurmlab/flo) with the curated 3D7 annotation provided at PlasmoDB (http://plasmodb.org, v32) as reference (12). The genome assembly and annotation are accessible under BioProject accession number PRJNA422809. Illumina sequencing data are deposited at the Sequence Read Archive under accession SRP154270. This Whole Genome Shotgun project has been deposited at DDBJ/ENA/GenBank under the accession QFXU00000000. The version described in this paper is version QFXU01000000.

**Whole genome alignments.** Whole genome alignment of the *P. falciparum* NF54 DiCre nuclear genome reported in this study and the wild-type *P. falciparum* 3D7 (13) and NF54 nuclear genome (14) were performed using ‘nucmer’ from the MUMMER package (v3.23) (15). Alignments with ≥ 99% similarity and longer 50 kb were plotted using ‘mummerplot’.

**Macrogamete assay.** Gametocyte cultures were produced as previously described, with small modifications. After gamete activation on day 14, the same 96 well plate used to measure exflagelletion was incubated over night at 28 degrees and analysed 24 hours after using an anti-Pfs25 antibody coupled to Cy3, by use of an Amersham CyDye monoclonal antibody labeling kit (GE Healthcare) (a kind gift from Andrea Ruecker and Michael delves from Imperial College, London). The labelled antibody was used at a 1:500 dilution of a 0.5-mg/ml IgG stock solution in the assay, as previously shown (16). Female gamete formation was recorded by single-image fluorescence microscopy using the x20 microscope magnification. Macrogametes were quantified by specific algorithms designed with the open-source ICY Image Analysis software (17) and the Mann-Whitney t test was used for calculating statistical significance P< 0.05.

**References**

1. Knuepfer E, Napiorkowska M, van Ooij C, Holder AA. 2017. Generating conditional gene knockouts in Plasmodium - a toolkit to produce stable DiCre recombinase-expressing parasite lines using CRISPR/Cas9. Sci Rep 7:3881.

2. Moon RW, Hall J, Rangkuti F, Ho YS, Almond N, Mitchell GH, Pain A, Holder AA, Blackman MJ. 2013. Adaptation of the genetically tractable malaria pathogen Plasmodium knowlesi to continuous culture in human erythrocytes. Proc Natl Acad Sci U S A 110:531-6.

3. Birnbaum J, Flemming S, Reichard N, Soares AB, Mesen-Ramirez P, Jonscher E, Bergmann B, Spielmann T. 2017. A genetic system to study Plasmodium falciparum protein function. Nat Methods 14:450-456.

4. Thomas JA, Collins CR, Das S, Hackett F, Graindorge A, Bell D, Deu E, Blackman MJ. 2016. Development and Application of a Simple Plaque Assay for the Human Malaria Parasite Plasmodium falciparum. PLoS One 11:e0157873.

5. Chin CS, Alexander DH, Marks P, Klammer AA, Drake J, Heiner C, Clum A, Copeland A, Huddleston J, Eichler EE, Turner SW, Korlach J. 2013. Nonhybrid, finished microbial genome assemblies from long-read SMRT sequencing data. Nat Methods 10:563-9.

6. Boetzer M, Pirovano W. 2014. SSPACE-LongRead: scaffolding bacterial draft genomes using long read sequence information. BMC Bioinformatics 15:211.

7. Boetzer M, Pirovano W. 2012. Toward almost closed genomes with GapFiller. Genome Biology 13.

8. Chaisson MJ, Tesler G. 2012. Mapping single molecule sequencing reads using basic local alignment with successive refinement (BLASR): application and theory. BMC Bioinformatics 13.

9. Langmead B, Salzberg SL. 2012. Fast gapped-read alignment with Bowtie 2. Nat Methods 9:357-9.

10. Walker BJ, Abeel T, Shea T, Priest M, Abouelliel A, Sakthikumar S, Cuomo CA, Zeng Q, Wortman J, Young SK, Earl AM. 2014. Pilon: an integrated tool for comprehensive microbial variant detection and genome assembly improvement. PLoS One 9:e112963.

11. Soorni A, Haak D, Zaitlin D, Bombarely A. 2017. Organelle_PBA, a pipeline for assembling chloroplast and mitochondrial genomes from PacBio DNA sequencing data. BMC Genomics 18.

12. Bahl A, Brunk B, Crabtree J, Fraunholz MJ, Gajria B, Grant GR, Ginsburg H, Gupta D, Kissinger JC, Labo P, Li L, Mailman MD, Milgram AJ, Pearson DS, Roos DS, Schug J, Stoeckert CJ, Jr., Whetzel P. 2003. PlasmoDB: the Plasmodium genome resource. A database integrating experimental and computational data. Nucleic Acids Res 31:212-5.

13. Gardner MJ, Hall N, Fung E, White O, Berriman M, Hyman RW, Carlton JM, Pain A, Nelson KE, Bowman S, Paulsen IT, James K, Eisen JA, Rutherford K, Salzberg SL, Craig A, Kyes S, Chan MS, Nene V, Shallom SJ, Suh B, Peterson J, Angiuoli S, Pertea M, Allen J, Selengut J, Haft D, Mather MW, Vaidya AB, Martin DM, Fairlamb AH, Fraunholz MJ, Roos DS, Ralph SA, McFadden GI, Cummings LM, Subramanian GM, Mungall C, Venter JC, Carucci DJ, Hoffman SL, Newbold C, Davis RW, Fraser CM, Barrell B. 2002. Genome sequence of the human malaria parasite Plasmodium falciparum. Nature 419:498-511.

14. Bryant JM, Baumgarten S, Lorthiois A, Scheidig-Benatar C, Claës A, Scherf A. 2018. De Novo Genome Assembly of a Plasmodium falciparum NF54 Clone Using Single-Molecule Real-Time Sequencing. Genome Announc 6:e01479-17.

15. Kurtz S, Phillippy A, Delcher AL, Smoot M, Shumway M, Antonescu C, Salzberg SL. 2004. Versatile and open software for comparing large genomes. Genome Biol 5:R12.

16. Delves MJ, Miguel-Blanco C, Matthews H, Molina I, Ruecker A, Yahiya S, Straschil U, Abraham M, Leon ML, Fischer OJ, Rueda-Zubiaurre A, Brandt JR, Cortes A, Barnard A, Fuchter MJ, Calderon F, Winzeler EA, Sinden RE, Herreros E, Gamo FJ, Baum J. 2018. A high throughput screen for next-generation leads targeting malaria parasite transmission. Nat Commun 9:3805.

17. de Chaumont F, Dallongeville S, Chenouard N, Herve N, Pop S, Provoost T, Meas-Yedid V, Pankajakshan P, Lecomte T, Le Montagner Y, Lagache T, Dufour A, Olivo-Marin JC. 2012. Icy: an open bioimage informatics platform for extended reproducible research. Nat Methods 9:690-6.
