# Supplementary material for: A Novel Tool for the Generation of Conditional Knockouts To Study Gene Function across the Plasmodium falciparum Life Cycle
Source: mBio. 2019 Sep 17;10(5):e01170-19. doi: 10.1128/mBio.01170-19 (PMC6751054; doi:10.1128/mBio.01170-19)
Supplement: TABLE S2 [file mBio.01170-19-st002.docx]

**Table S2**

| Name | Sequence |
| --- | --- |
| FIKK7.1HRForward | GTAACAGACTTAGGAGGAGATCTGGTTTACTAGAATTCGAACAAATTCTTG |
| FIKK7.1HRReverse | CCATATATTACATCTGTTTTATACATTAACTGAAGTCTTTTACTACTTTTAGAATAAC |
| T2a-NeoForward: | GTATCCATACGATGTACCGGATTATGCGGTCGTCGACGGAGAAGGAAGAGGAAGTTTATTAACATGTGG |
| T2a-NeoReverse: | CTATACGAAGTTATTGTATATTATTTTTTTTATTTACCTCGAGTTAGAAGAACTCGTCAAGAAGGCGATAGAAGGC |
| BglII-AMA1-F | GCTAACGTAACAGACTTAGGAGGAGATCTCCAACAGAACCTCTTATGTCACC |
| AMA1-R2 | AATTTTCATTTTATCATAAGTTGGTTTATGTTCAGG |
| AMA1-ILI-F2 | CAACTTATGATAAAATGAAAGTAAATAAAAAAAATAATATACAATAACTTC |
| AMA1rec-ILI-R2 | GCAGATGAGGCAATTATTATCTAAAAGAATATAAAATATATAAATATATATATATATAC |
| FIKK7.1RCForward: | GTTATTCTAAAAGTAGTAAAAGACTTCAGTTAATGTATAAAACAGATGTAATATATGG |
| FIKKUniversalReverse: | CTCTTCCTTCTCCGTCGACGACCGCATAATCCGGTACATCGTATGGATACGAGCCAG |
| FIKK7.1 Geneart | GTGTTAATTATTATGATTTTAATTTAATATCAATTCCAACTATTGGTTATTCTAAAAGTAGTAAAAGACTTCAGTTAATGTATAAAACAGATGTAATATATGGGGAAAATGAGGTAAATAAAAAAAATAATATACAATAACTTCGTATAGCATACATTATACGAAGTTATTATATATGTATATATATATATATTTATATATTTTATATTCTTTTAGAACGACAAAAATAATCTGAAAAAGAAGAAACTTTTCCTGAAAAAAGTCCCTGCAAATTTATGGATCGAACAGTACAAACTTATGAAAGAATACGATGGTGAATATGTGTATAGCGGCGAAAACTACGTTATGGAATTTTTGGTCTTAAGTTTCCTGGATACATACCACCCGAATATTTGTCCTAAATTGTACAAAATTCTTTACGAACCGCCAAACAAAGAATATATTAAGGATGAAAATAAGAAATTCCAGAATATTGATGATTTTGTCAAATATATGGAAGACATTATCGAGCACAATAAACGTAATAATGCAAACAACAACGTGGATAACAATAACAATATTCACAACCATAAAAACAACATCAACTATTGCATTACAAATAGCGACAACAAGCATGACAACAATAACAACGAAACAGCGACAACAACTGTGGGTACGTGGTAATGGTGAGCGAATATTACGGTGAGGATATCTTTGACTTTATCATTAAACGTCGTAAGAATATCTTCTTAAAAATTCGGCGCAAAGACAAAATTAATATTTTACATGCCTGCTTAAAACTGCTGGCCCGGCTGCATGACGCCGGACTGTGTCATCTGGATTTAACACCAGACAACATTCTGATTAGCAAATCCATGGACCTGCGCTTATGTGACTTCGCGAAATCGACACCGATGTATAGTAACAAATTACGCCACTTAAAAGAATCCGAAGACAGTTATAAGTTCGAAAGTTATGAGACGCACGTGGCTAAATCAGCCTACACCCCCCCGGAATGTTGGGAAATTTACTGGCGCTACTATGAACTGAAGATTAAGGAACCGTTGGAATATCTCAAGCTGATTACCAATCAGGAAGAACGTAAACAGTTCTATTTTGACGTCGCATGCGCGGATAAATTTATGCTGGGCGTCCTTTTCATCTGGATCTGGACTTCCGGGAATTTGTGGGTCTGTTCAGATCCGCTGCAAGATGACTATTTTCACTGTCTCATGAAGTCTGATATGAACTTTAATAACTTCCCTTGCTCACAAAATTGGCCGCATGGGTTAAAACATATCATTAAGCAGTTACTGCATATGAAGTACCGCAAAGACCTGAATCTGAACATCCTGGGGATCCACCCGTGGTGGTATAAAAAAAAAATGTACCCATATGATGTACCGGATTACGCAGGTTATCCGTATGACGTTCCGGACTATGCTGGCTCGTATCCATACGATGTACCGGATTATGCGGTCTAACTCGAGGTAAATAAAAAAAATAATATACAATAACTTCGTATAGCATACATTATACGAAGTTATTATATATGTATATATATATATATTTATATATTTTATATTCTTTTAGATGAGTAAAGGAGAAGAACTTTTCACTGGAGTTGTCCCAATTCTTGTTGAATTAGATGGTGATGTTAATGGGCACAAATTTTCTGTCAGTGGAGAGGGTGAAGGTGATGCAACATACGGAAAACTTACCCTTAAATTTATTTGCACTACTGGAAAACTACCAGTTC |
| RecAMA1-HA-SalI | ATAATAATTGCCTCATCTGCCGCCGTGGCTGTGCTTGCCACCATATTGATGGTATACCTTTATAAAAGGAAAGGGAACGCCGAAAAATACGATAAAATGGACGAACCCCAAGACTATGGGAAATCAAATTCAAGAAATGACGAAATGCTTGATCCTGAAGCATCATTTTGGGGGGAAGAAAAAAGGGCATCACACACAACACCCGTACTTATGGAAAAACCATATTACTACCCATATGATGTACCGGATTACGCAGGTTATCCGTATGACGTTCCGGACTATGCTGGCTCGTATCCATACGATGTACCGGATTATGCGGTCG/TCGACGGAGAAGGAAGAGGAAGT |
| synAMA1-F2 | ATAATAATTGCCTCATCTGCCGC |
| HD126 | ACTTCCTCTTCCTTCTCCGTCGACGACCGCGTAATCCGGTAC |
